# Decentralized multi-agent reinforcement learning based on best-response policies

**DOI:** 10.3389/frobt.2024.1229026

**Published:** 2024-04-16

**Authors:** Volker Gabler, Dirk Wollherr

**Affiliations:** Chair of Automatic Control Engineering, TUM School of Computation, Information and Technology, Technical University of Munich, Munich, Germany

**Keywords:** multi-agent reinforcement learning, game theory, deep learning, artificial intelligence, actor–critic algorithm, multi-agent, Stackelberg, decentralized learning schemes, reinforcement leaning

## Abstract

**Introduction:** Multi-agent systems are an interdisciplinary research field that describes the concept of multiple decisive individuals interacting with a usually partially observable environment. Given the recent advances in single-agent reinforcement learning, multi-agent reinforcement learning (RL) has gained tremendous interest in recent years. Most research studies apply a fully centralized learning scheme to ease the transfer from the single-agent domain to multi-agent systems.

**Methods:** In contrast, we claim that a decentralized learning scheme is preferable for applications in real-world scenarios as this allows deploying a learning algorithm on an individual robot rather than deploying the algorithm to a complete fleet of robots. Therefore, this article outlines a novel actor–critic (AC) approach tailored to cooperative MARL problems in sparsely rewarded domains. Our approach decouples the MARL problem into a set of distributed agents that model the other agents as responsive entities. In particular, we propose using two separate critics per agent to distinguish between the joint task reward and agent-based costs as commonly applied within multi-robot planning. On one hand, the agent-based critic intends to decrease agent-specific costs. On the other hand, each agent intends to optimize the joint team reward based on the joint task critic. As this critic still depends on the joint action of all agents, we outline two suitable behavior models based on Stackelberg games: a game against nature and a dyadic game against each agent. Following these behavior models, our algorithm allows fully decentralized execution and training.

**Results and Discussion:** We evaluate our presented method using the proposed behavior models within a sparsely rewarded simulated multi-agent environment. Although our approach already outperforms the state-of-the-art learners, we conclude this article by outlining possible extensions of our algorithm that future research may build upon.

## 1 Introduction

Based on recent advances in robotics research over the last few decades, automated robotic systems have been established in everyday life, even beyond industrial applications. Nonetheless, it remains tedious and challenging to impart new tasks to robots, especially if the environment is stochastic and hard to model. In this context, applied machine learning (ML), specifically reinforcement learning (RL), is a promising research field that aims to continuously improve robotic performance from collected task trial samples. In particular, the core motivation is to equip robots with the ability to explore and learn unknown tasks simultaneously without relying on an accurate model of the environment or the task. Building upon this, the concept of MARL has raised interest in improving scalability by executing tasks by a fleet of robots rather than a single autonomous unit. In order to exploit results from single-agent RL, a common paradigm in MARL is *centralized learning with decentralized execution*. Nonetheless, it is desirable to handle each robot as an independent individual, such that the learning phase of a MARL algorithm also scales well. In contrast to simulated environments, where access to other agents’ policies and observation is realistic, this assumption is overly restrictive for real robot systems and adds additional constraints to heterogeneous robot fleets.

Therefore, the contribution of this article is a novel AC method for cooperative MARL problems in sparsely rewarded environments. Our MARL algorithm allows fully decoupling learning among the agents while achieving comparable performance to current state-of-the-art MARL approaches. This approach uses the best-response policies of other agents conditioned on each agent’s policy output. This leverages the need to access the exact agent policies during centralized learning to achieve a fully decentralized learning scheme. This decision-theoretic principle stems from Stackelberg equilibria from game theory and is tailored to non-zero-sum games in the scope of this article.

Our proposed method incorporates another multi-robot planning and game theory concept by explicitly differentiating between *interactive* task rewards and agent-specific costs, i.e., *native* costs. In other words, each agent estimates the performance of the joint policy with regard to the current task to be learned but also a cost critic that evaluates agent-specific costs.

In the remainder of this article, we briefly summarize the state-of-the-art algorithms of MARL in Section 2, followed by a summary of the technical foundations of this article and the technical problem in Section 3. The core concept of our proposed framework is outlined in Section 4. In order to evaluate the presented method, we present the collected results of our method compared against state-of-the-art MARL algorithms in Section 5. Based on these results, we discuss our algorithm and explicitly sketch conceptual modifications of our approach in Section 6. Eventually, we conclude this article in Section 7.

## 2 Related work

Even though early applications of RL in robotic systems have shown promising results (Ng et al., 2004; Kolter and Ng, 2009), it was the success of outperforming humans in computer games via deep RL (Mnih et al., 2015; Silver et al., 2016; Vinyals et al., 2019) without suffering from catastrophic interference (McCloskey and Cohen, 1989) problems that has opened the door for RL applications within complex, real-world environments. Given the computational power of modern graphics processing units (GPUs), policy gradients such as the stochastic policy gradient from Sutton et al. (1999a) or the deterministic policy gradient (PG) from Silver et al. (2014) have been realized via function approximators such as neural networks (NNs). A famous example is given as the deep deterministic policy gradient (DPG) by Lillicrap et al. (2016). DDPG has shown that deep RL can also be applied on continuous action spaces such that the applicability of RL within robotic systems has been boosted drastically ever since. Even though further PG methods have been developed in order to improve the variance sensitivity issue, such as trust region policy optimization (Schulman et al., 2015), proximal policy optimization (Schulman et al., 2017), or maximum *a posteriori* policy optimization (Song et al., 2020), the majority of algorithms relies on an AC architecture, where an additional critic reduces the variance drastically, such as the soft actor–critic (SAC) (Haarnoja et al., 2018). As an intense outline of advances in single-agent RL is beyond the scope of this article, we encourage the interested reader to available literature survey papers (Kaelbling et al., 1996; Kober et al., 2013; Arulkumaran et al., 2017). Building upon the results from single-agent RL, MARL has gained great interest over the last decades and is thus outlined separately in the following.

### 2.1 Multi-agent reinforcement learning

In addition to solving complex Markov decision process (MDP) problems, the decentralized extension of the Markov game (MG) has gained attention in the context of MARL (van der Wal, 1980; Littman, 1994). The naive approach of extending Q-learning to a set of $N_{A}$ independent learners (Tan, 1993) works well for small-scale problems or selective applications. Similar to deep RL, initial results on MARL have been found on discrete action sets, such as the differentiable inter-agent learning by Foerster et al. (2016) or the explicit communication learning by Havrylov and Titov (2017); Mordatch and Abbeel (2017). In general, however, independent learners violate the Markov assumption (Laurent et al., 2011).

Multi-agent deep deterministic policy gradient (DDPG) is an extension of DDPG to MARL (Lowe et al., 2017), which also applies an AC architecture. During training, a centralized critic uses additional information about the other agents’ states and actions to approximate the Q -function. Given this centralized critic, each agent updates a policy that is only conditioned on the local observations of each agent. Thus, the actor only relies on local observations during execution. MADDPG has achieved very robust results in simulated benchmark environments (Mordatch and Abbeel, 2017) for cooperative and competitive scenarios. Various extensions to the multi-agent deep deterministic policy gradient (MADDPG) have been proposed. Li et al. (2019) introduced an extension to MADDPG that uses the minimax concept of game theory to make decisions under uncertainty. The idea is to take the best action in the worst possible case.

As pointed out by Ackermann et al. (2019), the overestimation bias is also present in MARL. Some initial works have proposed to bridge concepts from the single-agent domain (van Hasselt, 2010) to MARL (Sun et al., 2020). Thus, SAC has been adjusted to the multi-agent domain by Wei et al. (2018), for which further extensions have been outlined, e.g., Zhang et al. (2020) proposed a Lyapunov-based penalty term to the policy update to stabilize the policy gradient. As centralized learning inherently suffers from poor scaling, Iqbal and Sha (2019) introduced attention mechanisms in the multi-actor-attention-critic (MAAC). In order to cope with large-scale MARL, Sheikh and Bölöni (2020) explicitly differentiated between local and global reward metrics that each agent obtains from the environment.

In contrast to single-agent systems, the critic also suffers from the non-stationarity of the policies of other agents. This initiated the research on explicitly modeling the learning behavior of other agents, such as Foerster et al. (2018). Alternatively, Tian et al. (2019) proposed to model the MARL problem as an inference problem, i.e., to estimate the most likely action of the other agents and respond with the best response (br). Jaques et al. (2019) reversed this idea by applying counterfactual reasoning and thus incorporating the mutual influence among agents into the reward of each agent. They outlined a decentralized version of their algorithm, which applies behavioral cloning similarly to the decentralized version of MADDPG.

As a complete survey of MARL is beyond the scope of this article, we refer to Zhang et al. (2021), Hernandez-Leal et al. (2020), Yang and Wang (2020), Hernandez-Leal et al. (2019), and Nguyen et al. (2020) for a more detailed literature review. In order to illustrate the relevance of MARL from an application-driven perspective, there exists a variety of recent examples, such as logistics (Tang et al., 2021), the Internet of Things (Wu et al., 2021), or motion-planning for robots (He et al., 2021).

## 3 Preliminaries

As the methods presented in this article build upon various findings from the literature, we provide an insight into these methods. We begin with sketching the notation used in this article, followed by an initial example in the form of introducing MGs.

### 3.1 Notation

In order to outline the notation for the remainder of this article, we use p as an arbitrary placeholder variable. We denote $p^{\left(i\right)}$ as a variable explicitly assigned to agent *i*, while ${\left(p\right)}_{i\in N_{A}}$ denotes the *joint* team analog of the said variable for all agents. This is most commonly denoted as $\underline{p}$ for brevity, while ${\underline{p}}^{\left(-i\right)}$ denotes all elements of ${\left(p\right)}_{i\in N_{A}}$ except $p^{i}$ . Furthermore, we denote the vector as p and matrices as p , while $1^{p}$ , $1^{p\times p}$ , $0^{p}$ , and $0^{p\times p}$ denote identity- and zero-vectors/matrices. In general, we denote time-variant variables as $p_{t}$ , while a temporal successor $p_{t+1}$ is denoted as ′ for brevity. In the context of stochastic variables, we denote probability density functions (PDFs) as P[p] and conditionally dependent PDFs as $P\left[p_{1}|p_{2}\right]$ . Similarly, $E_{p_{1}∼\rho \left(p_{2}\right)}\left[\right]$ and $V{ar}_{p_{1}∼\rho \left(p_{2}\right)}\left[\right]$ symbolize the expectation and variance of the random variable $p_{1}$ that follows a probability distribution function *ρ*(), which depends on $p_{2}$ . Eventually, we denote hierarchical systems by denoting layer *k* as p{k} .

### 3.2 Markov game

**FIGURE 1:**
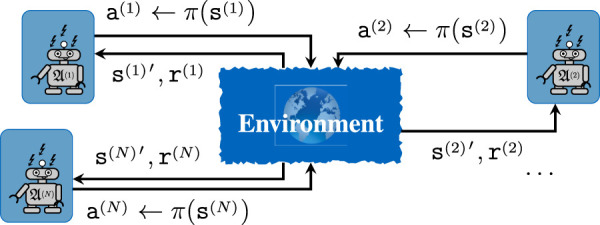
Sketch of a general MARL problem, where $N_{A}$ agents interact with each other in an unknown environment. Each agent has access to the individual state observation $s^{\left(i\right)}$ , which can be mapped to an action $a^{\left(i\right)}$ via the policy *π* ^(*i*)^ in such a manner that the expected individual return $r^{\left(i\right)}$ is maximized.

An MG is an extension of an MDP to the multi-agent domain, which is fully described by the tuple $\left(\underline{A},\underline{S},\underline{A},T,\underline{R},\gamma \right)$ , where $N_{A}$ agents $\underline{A}=\left(A^{\left(1\right)},A^{\left(2\right)},\ldots ,A^{\left(N_{A}\right)}\right)={\left(A\right)}_{i\in N_{A}}$ interact with each other in a stochastic environment (Shapley, 1952; Shapley, 1953), as shown in Figure 1. The state $\underline{s}=\left(s^{\left(1\right)},s^{\left(2\right)},\ldots ,s^{\left(N_{A}\right)}\right)\in \underline{S}$ of the environment with state space $\underline{S}$ is perceived as the individual state observations $s^{\left(i\right)}$ for each agent. Due to the Markov property, the dynamics of an MG is given by each individual choosing an action $a^{\left(i\right)}\in A^{\left(i\right)}\subset \underline{A}$ out of an agent-specific action space $A^{\left(i\right)}$ , thus forming a joint action $\underline{a}$ that transitions $\underline{s}$ to ${\underline{s}}^{\prime }$ according to a transition probability function $T:=P\left[{\underline{s}}^{\prime }|\underline{s},\underline{a}\right]$ , and $P\left[{\underline{s}}^{\prime }|\underline{s},\underline{a}\right]$ is the conditional probability for ${\underline{s}}^{\prime }$, given $\underline{s}$ and $\underline{a}$ . The individual reward functions $\underline{R}={\left(R^{\left(i\right)}:S^{\left(i\right)}\times A^{\left(i\right)}\times {\underline{A}}^{\left(-i\right)}\times S^{\left(i\right)}\to R\right)}_{i\in N_{A}}$ map a transition from $\underline{s}$ to ${\underline{s}}^{\prime }$, given $\underline{a}$ , to a numeric value for each agent $A^{\left(i\right)}$ , which is denoted as $r^{\left(i\right)}:{=R}^{\left(i\right)}\left(s^{\left(i\right)},a^{\left(i\right)},\underline{a^{\left(-i\right)}},s^{\left(i\right)\prime }\right)$ . Given this, each agent $A^{\left(i\right)}$ follows the stochastic behavior policy $a^{\left(i\right)}∼\pi^{\left(i\right)}\left(s^{\left(i\right)}\right)$ that intends to maximize the objective for each agent

$$\begin{array}{cc} J^{\left(i\right)} & :=\overset{\infty }{\underset{t=0}{\sum }}\gamma^{t}\int_{\underline{A}}\underline{\pi }\left({\underline{a}}_{t}|{\underline{s}}_{t}\right)\int_{\underline{S}}T\left({\underline{s}}_{t+1}|{\underline{s}}_{t},{\underline{a}}_{t}\right)\;r^{\left(i\right)}d{\underline{s}}_{t+1}d{\underline{a}}_{t}\\ & \;=\overset{\infty }{\underset{t=0}{\sum }}\gamma^{t}E_{{\underline{a}}^{\left(-i\right)}∼{\underline{\pi }}^{\left(-i\right)}\left(s_{t}^{\left(i\right)}\right)}\\ & \;\times \left[\int_{\underline{A}}\pi \left(a_{t}^{\left(i\right)}|s_{t}^{\left(i\right)}\right)P\left[{\underline{a}}^{\left(-i\right)}\right]\int_{\underline{S}}T\left({\underline{s}}_{t+1}|{\underline{s}}_{t},{\underline{a}}_{t}\right)r^{\left(i\right)}d{\underline{s}}_{t+1}d{\underline{a}}_{t}\right] \end{array}\;,$$ (1)

where the hyperparameter $\gamma \in \left(0,1\right]$ is a temporal decay weight that scales short-term versus long-term impact. In order to solve Equation 1, the state-value function,

$$V_{\underline{\pi }}^{\left(i\right)}\left(\underline{s}\right)=\overset{\infty }{\underset{t=0}{\sum }}E_{{\underline{a}}_{t}∼\rho \left(\underline{\pi }\right),\left({\underline{s}}_{t},{\underline{s}}_{t+1}\right)∼\rho \left(T,\underline{\pi }\right)}\left[\gamma^{t}r^{\left(i\right)}|{\underline{s}}_{0}\right],$$ (2)

the state-action value function,

$$Q_{\underline{\pi }}^{(i)}\left(\underline{s},\underline{a}\right)=r^{\left(i\right)}+\gamma E_{{\underline{s}}^{\prime }∼\rho \left(T,\underline{\pi }\right)}\left[V_{\underline{\pi }}^{\left(i\right)}\left({\underline{s}}^{\prime }\right)\right],$$ (3)

and the advantage function,

$$A_{\underline{\pi }}^{\left(i\right)}\left(\underline{s},\underline{a}\right)=Q_{\underline{\pi }}^{\left(i\right)}\left(\underline{s},\underline{a}\right)-V_{\underline{\pi }}^{\left(i\right)}\left(\underline{s}\right)$$ (4)

have been introduced as the multi-agent version of the Bellman backup operator for MDPs (Bellman, 1957). Given that the agents follow a fixed and optimal policy ${\underline{\pi }}^{*}$ , the dynamic programming problem eventually solves Equation 1 as the global optimum of the MG, as shown by Littman (1994). Given the optimal $Q_{{\underline{\pi }}^{*}}$ function, the optimal policies for each agent can be obtained as the following:

$$\pi^{\left(i\right)}{\left(\underline{s}\right)}^{*}\leftarrow \arg\underset{\pi^{\left(i\right)}}{\max}{\left.Q_{{\underline{\pi }}^{*}}^{\left(i\right)}\left(\underline{s},{\underline{a}}^{\left(-i\right)},a\right)\right|}_{a\leftarrow \pi^{\left(i\right)}\left(\underline{s}\right)}.$$ (5)

As solving Equation 5 requires each agent to follow an optimal policy, the definition of a best-response policy is of importance in MGs.

Definition 3.1 Best response policy

Given a joint policy ${\underline{\pi }}^{\left(-i\right)}$ for the neighboring agents of agent $A^{\left(i\right)}$ , a policy ^br^ *π* ^(*i*)^ is called a br to ${\underline{\pi }}^{\left(-i\right)}$ if and only if

J(i)a(i)br←π(i)br|π_(−i)≥J(i)a(i)≠a(i)br|π_(−i),

i.e., the agent $A^{\left(i\right)}$ cannot improve the individual payoff return $J^{\left(i\right)}$ by deviating from π(i)br (Shoham and Leyton-Brown, 2008).

Within an MG, the optimal policy requires that the policies of the individual agents are the br to the policies of the surrounding agents, leading to the definition of a Nash equilibrium (NE).

Definition 3.2 Nash equilibrium

According to Nash (1950), a policy π_NE:=πNEi∈NA is a NE if and only if each agent following πiNE∈π_NE results in each policy being a br policy, according to Definition 3.1. Replacing the objectives $J^{\left(i\right)}$ by the state-action value $Q^{\left(i\right)}$, this requires

Qπ(i)NE,π_(−i)NE(i)≥Qπ~(i),π_(−i)NE(i)Qπ(i)NE,π_(−i)NE(i)≥QπNE(i),π_~(−i)(i)with π~≠πNE,∀A(i)∈A_,∀s_∈S_,

to hold on the global state space $\underline{S}$ .

Nonetheless, in real-world problems, neither ${\underline{\pi }}^{*}$ nor the value functions are known. In addition, the environment is characterized by multiple learners, whose policies and, thus, actions vary over time and cannot directly be controlled by an individual agent in an MG. This results in the problem formulation of this article.

### 3.3 Multi-agent reinforcement learning problem

Given a set of agents ${\left(A\right)}_{i\in N_{A}}$ that try to optimize their individual accumulated discounted reward, according to Section 3.2, an optimal policy for each agent has to be found, which fulfills the following:

• ${\left(\pi \leftarrow \arg\max Q_{\pi }\right)}_{i\in N_{A}}$ according to Equation 5.
• The joint action $\underline{a}={\left(a\leftarrow \pi^{*}\right)}_{i\in N_{A}}$ is an NE of the MG, according to Definition 3.2.

We will continue with a short overview of RL methods that have been established as the current state-of-the-art methods within single-agent RL and MARL.

### 3.4 Policy gradient methods

Obtaining an optimal policy *π*_II_, parameterized by II, has been tackled by generating PGs (Sutton et al., 1999b) that estimate the stochastic gradient over II of a policy, and the policy-loss function is defined as the following:

$$\nabla_{\Pi }J\left(\pi_{\Pi }\right)=E_{s∼\pi \left(s\right)}\left[\overset{\infty }{\underset{t=0}{\sum }}\nabla_{\Pi }\log\pi_{\Pi }\left(a_{t}|s_{t}\right)\;\chi_{t}\right],$$ (6)

where $\chi_{t}$ may, for example, be the single agent version of Equation 3 or Equation 4, i.e., $Q_{\pi }$ or $A_{\pi }$ . If one can obtain the gradient $\nabla_{a}\chi_{t}$ directly, i.e., the action space is continuous and the environment is stationary, it is also possible to obtain the deterministic policy gradient (DPG) from Equation 6 as the following:

$$\nabla_{\Pi }J\left(\pi_{\Pi }\right)=E_{s∼D}\left[\nabla_{\Pi }\pi_{\Pi }\left(a|s\right){\left.\nabla_{a}\chi \right|}_{a\leftarrow \pi \left(s\right)}\right],$$ (7)

where the expectation is approximated by drawing samples from an experience replay buffer D that contains observed environment transitions. Exemplary DDPG uses $\chi_{t}:=Q_{\pi }$ in order to obtain the gradient of the state-action value in Equation 7. As it can be seen in Equations 6 and 7, PGs and DPGs are generally highly sensitive to the variance of $\chi_{t}$ . As a consequence, AC methods have been outlined that add a policy evaluation metric to the policy update of PG methods.

### 3.5 Actor–critic methods

As the accumulated reward does generally suffer from high variance over repeated episodes, AC algorithms simultaneously estimate $A_{\pi }$ or $Q_{\pi }$ alongside the PGs in Equation 6. The deep Q-network presented by Mnih et al. (2015) uses NNs as function approximators, thus approximating $Q_{\pi }$ by $Q_{\Theta }$ and Q†Θ , parametrized by Θ, where Q† denotes the *target-net* of Q . These two function approximators are then used to learn $Q_{\pi }$ via off-policy temporal-difference learning, which is obtained via iteratively minimizing the loss function as follows:

LQΘ:=Es,a,r,s′∼D12p−QΘs,a2withp=rs,a,s′+γ1−dV†Θs′V†Θs′=Ea′←πs′Q†Θs′,a′, (8)

where D is again a replay buffer that stores experienced transitions from the environment during the exploration process. Each sample contains the state s , action a , next state $s^{\prime }$ , the experienced reward r, and the termination flag d . The term (1−d) thus ignores the value of the successor state in the Bellman backup operator in Equation 3 at the terminal states.

The SAC (Haarnoja et al., 2018) is an extension of the general AC that approximates the solution of Equation 1 via a maximum entropy objective by introducing a soft-value function, thus replacing Equation 2 by the following:

$$\begin{array}{cc} V_{\pi }\left(s\right) & :=E_{\left(s_{t},a_{t},s_{t+1}\right)∼D}{\left[\overset{\infty }{\underset{t=0}{\sum }}\gamma^{t}\left(r\left(s_{t},a_{t},s_{t+1}\right)+\alpha H\left(\pi \left(\cdot |s_{t}\right)\right)\right)\right]}_{s_{0}=s}, \end{array}$$ (9)

In Eq. 9 H(⋅) denotes the policy entropy at a given state and *α* is a temperature parameter that weighs the impact of the entropy against the environment reward. In contrast to Equation 2, this objective explicitly encourages exploration in regions of high rewards, thus decreasing the chance of converging to local minima. Furthermore, two function approximators are used for the critic as in the twin-delayed deep deterministic policy gradient (TD3) such that the target value function in Equation 8 is obtained as the following:

V†Θs′=Ea′∼πs′minj=1,2Q†Θ,js′,a′−α⁡logπΠa′|s′, (10)

In Eq. 10 a is obtained from $\pi \left(s^{\prime }\right)$ , whereas $s^{\prime }$ is drawn from D . In contrast to this, the actual policy loss is obtained by applying the *reparameterization trick* as follows:

LπSACΠ:=Es∼Dminj=1,2Q†Θ,js,fΠs,ζ⏟χ.−α⁡logπΠfΠs,ζ|s, (11)

which computes a deterministic function $f_{\Pi }\left(s,\zeta \right)$ that depends on the state s , policy parameters Π, and independent noise vector *ζ* drawn from a fixed distribution, e.g., mean free Gaussian noise. In contrast to DDPG, this parameterized policy is also squashed via a tanh function to the bounds of the action space, thus resulting in valid samples that can be used to generate a stochastic policy for the stochastic policy gradient update step.

### 3.6 Multi-agent actor–critic algorithms

The methods mentioned above have been recently extended to the multi-agent domain. The MADDPG extends AC with DDPG by proposing the schematic representation of decentralized execution in combination with centralized learning. As such, each $A^{\left(i\right)}$ learns an individual (deterministic) policy $\pi :=S^{\left(i\right)}\times A^{\left(i\right)}\mapsto \left[0,1\right]$ while setting $\chi_{t}:=Q^{\left(i\right)}\left(\underline{s},\underline{a}\right)$ in Equation 7, which has access to the observations $s^{\left(i\right)}$ , actions $a^{\left(i\right)}$, and policies of all agents such that Equation 8 can be directly applied to the multi-agent domain. This requires having access to all policies during learning in order to calculate the target values of Equation 8. Similar approaches have been proposed by MAAC and counterfactual multi-agent (COMA)-PG, which additionally incorporate a baseline value function for the policy update and thus use the multi-agent advantage function

$$\chi :=A^{\left(i\right)}\left(\underline{s},\underline{a}\right)=Q^{\left(i\right)}\left(\underline{s},a^{\left(i\right)},{\underline{a}}^{\left(-i\right)}\right)-V_{b}\left(\underline{s},{\underline{a}}^{\left(-i\right)}\right)$$ (12)

for their policy loss declarations. The baseline $V_{b}\left(\underline{s},{\underline{a}}^{\left(-i\right)}\right)$ estimates the value of a current state and the opponents' current actions such that optimizing Equation 12 leads to a best-response action of the agent $A^{\left(i\right)}$, according to Definition 3.1. Although COMA substitutes Equation 12 into Equation 6, MAAC uses SAC, thus inserting Equation 12 into Equation 11. Furthermore, MAAC improves the centralized critic by adding an attention mechanism that explicitly learns which parts of the observations have an actual impact on the values of the critic.

## 4 Technical approach

**FIGURE 2:**
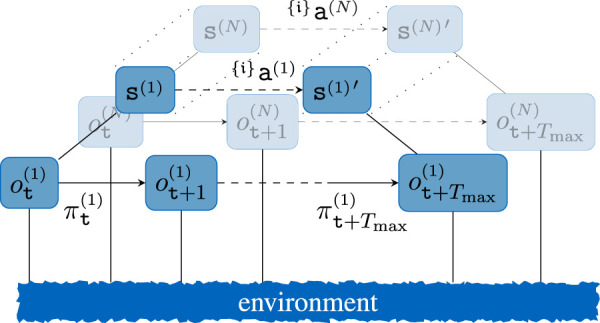
Exemplary step of a two-level hierarchical MARL step where each low-level step represents an interaction with the environment from Figure 1. For brevity, only selective nodes and edges are labeled. The upper layer acts synchronously, such that the observed transition would qualify for centralized learning for all layers, which is emphasized via the dashed lines for the upper layer.

A key challenge for real-world applications in multi-agent systems is the ability to handle decentralized decisions asynchronously. Although most MARL approaches allow decentralized execution, they still rely on centralized learning (Lowe et al., 2017). This imposes various constraints on the overall multi-agent system, e.g., the necessity to have access to all observations of all agents during learning. Furthermore, real robot systems are often commanded by a task planner or similar, where each robot is assigned a dedicated sub-task. Similarly, the upper layer could be realized via a hierarchical reinforcement learning (HRL) learner that learns to allocate tasks to each agent in a team-optimal manner. To visualize the necessity of our algorithm, a two-layered hierarchical decision framework for a multi-agent system is shown in Figure 2. The upper layer could either stem from a sub-task allocator that assigns tasks to each agent or a multi-agent HRL algorithm. As can be seen from this figure, centralized learning would not only require synchronous updates along all agents and layers, but it also would require knowing the current output of the upper layer of each agent. In order to leverage this constraint, we propose a novel decentralized MARL concept that builds upon the concept of best-response policies and separates joint rewards from internal agent objectives.

### 4.1 Decentralized MARL based on Stackelberg equilibria

In order to achieve a decentralized model for MARL problems, a previous work has evaluated the application of predicting the br policy to the inferred action of an opponent (Tian et al., 2019) or assuming overly restrictive access to the environment feedback of other agents. The latter is always fulfilled for centralized learning. In order to decouple the decentralized learning procedure, we propose a similar idea to that of Tian et al. (2019) and instead reformulate their inference-based policy by modeling the br policy of other agents. In detail, we apply the concept of Stackelberg equilibria. The Stackelberg equilibrium evaluates the br of an agent if the opponent has unveiled the current actions. Therefore, each agent regresses not only a policy ${\pi^{\left(i\right)}}_{\Pi }:=S^{\left(i\right)}\mapsto A^{\left(i\right)}$, parameterized by Π, that intends to optimize the player-individual agent-objective but also a br policy ${{\underline{\pi }}_{\text{br}}^{\left(-i\right)}}_{\Xi }:=S^{\left(i\right)}\times A^{\left(i\right)}\mapsto {\underline{A}}^{\left(-i\right)}$ − parameterized by Ξ − that represents the reactions of the other agent(s) at each step.

In addition to regressing the br policy of the other agent, we further claim that it is beneficial to distinguish between joint task rewards and individual or native cost terms. In general, we assume that the individual reward for a cooperative MARL problem is given in the following form:

$$\begin{array}{cc} J^{\left(i\right)}\left(\underline{s},\underline{\pi },{\underline{s}}^{\prime }\right)= & \overset{\infty }{\underset{t=0}{\sum }}\gamma^{t}E\left[r^{\left(i\right)}\left({\underline{s}}_{t},\underline{\pi },{\underline{s}}_{t+1}\right)-{{\hat{c}}_{\text{nat}}}^{\;\;\left(i\right)}\left({\underline{s}}_{t},\pi^{\left(i\right)},{\underline{s}}_{t+1}\right)\right] \end{array}\in R,$$ (13)

Thus, the individual reward in Eq. 13 consists of a joint or cooperative task reward that depends on the joint action or policy, as well as an interactive cost component that only affects each player. Although some existing work assumes to directly have access to local and global rewards, i.e., to obtain $r\left(\underline{s},\underline{\pi },{\underline{s}}^{\prime }\right)$ and ${{\hat{c}}_{\text{nat}}}^{\;\;\left(i\right)}$ directly (Sheikh and Bölöni, 2020), we propose a model that only has access to the agent reward and the averaged joint task reward of all agents tailored toward sparsely rewarded environments. Thus, the cost of the agents needs to be estimated from this joint reward at each transition. Thus, we apply the following:

$$\begin{array}{cc} {{\hat{c}}_{\text{nat}}}^{\;\;\left(i\right)}\left(s^{\left(i\right)},\underline{a},s^{\left(i\right)\prime }\right)\approx & \min\left(\frac{1}{N_{A}}\overset{N_{A}}{\underset{\begin{array}{c} j=1 \end{array}}{\sum }}r^{\left(j\right)}\left({\underline{s}}^{\left(j\right)},\underline{a},{\underline{s}}^{(j)\prime }\right)-r^{\left(i\right)}\left(s^{\left(i\right)},\underline{a},s^{\left(i\right)\prime }\right),0\right)\\ \approx & \min\left(\frac{1}{N_{A}}\overset{N_{A}}{\underset{\begin{array}{c} j=1 \end{array}}{\sum }}r^{\left(j\right)}-r^{\left(i\right)},0\right)\;\text{where}\;\underline{r}∼D^{\left(i\right)}, \end{array}$$ (14)

i.e., we keep the individual agent reward as the joint task reward, which solely depends on the observation of each agent. In addition, we propose to regress a non-negative auxiliary cost term that contains information about *local* interaction penalties for each agent. Exploiting the rare occurrence of costs within sparsely rewarded environments, we estimate the step cost for each agent by the difference of the average rewards of all agents compared to the individual agent reward. Having collected empirical data within a replay buffer $D^{\left(i\right)}$ for each agent, the numeric cost is approximated as a non-negative difference of the average reward values of all agents without the necessity of explicitly accessing the observations of each agent. Finally, our br-AC approach approximates the following:

• the (interactive) task critic $Q_{\text{int}}^{\left(i\right)}:=S^{\left(i\right)}\times \underline{A}\mapsto R$ , which intends to maximize the accumulated task reward.
• the (native) agent critic $Q_{\text{nat}}^{\left(i\right)}:=S^{\left(i\right)}\times A^{\left(i\right)}\mapsto R$ , which intends to minimize the agent-specific costs.

Therefore, the final goal of each agent is to maximize the interactive task critic, i.e., optimize the accumulated team reward, while minimizing the agent-specific cost penalties from the native agent critic. This concept is similar to the idea of combining RL rewards while also minimizing a myopic objective by means of numeric optimization *cf.* (Englert and Toussaint, 2016). The agent policies and critics can then be regressed by means of existing AC methods, such as SAC or TD3. In contrast to the default methods, the policies need to optimize the joint task critic and the native agent critic simultaneously. Therefore, the difference between the two critics provides the final critic that is used for the policy gradient of the current actor. Eventually, the br policy needs to be updated as well. In contrast to the agent policy, the native critic is independent of the br policy and can thus be neglected for the update of the br policies. As we emphasize on cooperative MARL, the br policies intend to optimize the joint task-critic as well. Thus, the br policy is found by obtaining the gradient of the joint task critic with regard to the policy of the other agents after applying the current agent policy. Denoting the cost estimation from Equation 14 as GetCost, a single update step for agent *i* is sketched in Algorithm 1. The dedicated critic losses are denoted as CriticLoss and CostCriticUpdate in Algorithm 1. The native cost critic is regressed by calculating

Algorithm 1 Decentralized br policy-based MARL update step for agent *i*. Due to the decentralized learning, the update step can be run in a fully parallelized procedure. For brevity, the exploration is omitted from this algorithm skeleton.

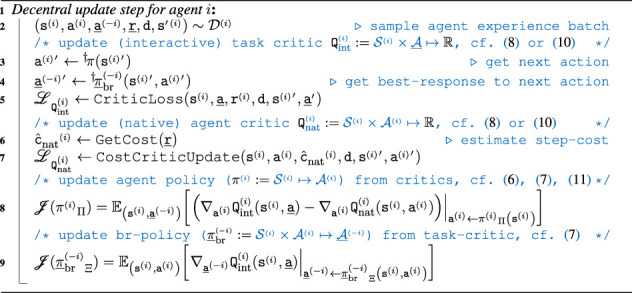

p=c^nat(i)+γ1−dmaxj=1,2Q†nat,j(i)s(i),a(i)′a(i)′←π(i)†s(i)′, (15)

in Equation 8. Nonetheless, it has to be noted that Equation 15 uses the maximum critic value to account for the overestimation bias as the cost critic intends to minimize the accumulated native costs. We explicitly do not apply SAC for the cost critic as exploration should be emphasized on the task to be learned rather than exploring accumulated costs. In order to regress the joint task critic, a similar approach to existing AC approaches is followed. Querying $a^{(i)\prime }$ identically to Equation 15, we set

p=r(i)s(i),a_,s(i)′+γ1−dminj=1,2Q†nat,j(i)s(i)′,a_′+pSACa(−i)′←π_†br(−i)Ξs(i)′,a(i)′pSAC=−α⁡logπ(i)a(i)′|s(i)′for an SAC model0else, (16)

in Equation 8.

For brevity, Algorithm 1 only sketches the update, i.e., learning, step of our proposed multi-agent reinforcement learning (MARL) approach. During the exploration phase, each agent samples from their own policy and stores the actions and rewards of the other agents. In other words, each agent stores a tuple of $\left(s^{\left(i\right)},\underline{a},\underline{r},d,s^{\left(i\right)\prime },\right)$ in the replay buffer $D^{\left(i\right)}$ until the end of an episode is reached or the task is completed. After collecting new data for a fixed number of episodes, Algorithm 1 is run for a fixed number of episodes.

Eventually, we distinguish between two interaction schemes in order to model ${\pi^{\left(-i\right)}}_{\Pi ,br}$ . First, the other agents can be modeled as an unknown black-box system, usually denoted as a *game-against-nature* within game theory. Thus, a single policy is tracked as follows:

$${{\underline{\pi }}_{\text{br}}^{\left(-i\right)}}_{\Xi }:=S^{\left(i\right)}\times A^{\left(i\right)}\mapsto {\left(\underline{A}\right)}_{j\in -i},$$ (17)

which models an interaction with the current agent and the *responsive* nature. Our second approach uses a dyadic interaction scheme and models the br policy of each agent to the current agent individually via Eq. 18.

$${{\underline{\pi }}_{\text{br}}^{\left(-i\right)}}_{\Xi }\leftarrow {\left({\pi_{\text{br}}^{\left(j\right)}}_{\Xi }:=S^{\left(i\right)}\times A^{\left(i\right)}\mapsto A^{\left(j\right)}\right)}_{j\in -i}.$$ (18)

This requires to regress $N_{A}-1$ opponent policies rather than a single policy in Equation 17. Both policies leverage the effect of a mutual interaction among the other agents to diminish combinatorial explosion.

## 5 Results

In this section, we evaluate the performance of our decentralized br policy MARL framework within the simulation environment from Appendix 1. Within this environment, we evaluated our algorithm against state-of-the-art algorithms within MARL, namely, MADDPG and multi-agent soft actor–critic (MASAC).

Given our adjusted multi-agent particle environment (MPE) as outlined in Appendix 1, we ran a decentralized version of TD3 (Ackermann et al., 2019) and the multi-agent version of Haarnoja (2018) for the joint critic in our algorithm. Given the dyadic and game-against-nature variants, we use the following notations:

• The state-of-the-art algorithms are directly denoted as commonly known in the literature, i.e., MADDPG and MASAC.
• Our extension of TD3 is denoted as br-TD3-dyad/nature.
• Our extension of SAC is denoted as br-SAC-dyad/nature.

As stated above, our main emphasis is set on improving the performance in sparsely rewarded environments. Furthermore, we explicitly tailor our approach to continuous action spaces in cooperative sections. Therefore, we applied the cooperative collection task, according to the parameterization in Appendix 2.

**FIGURE 3:**
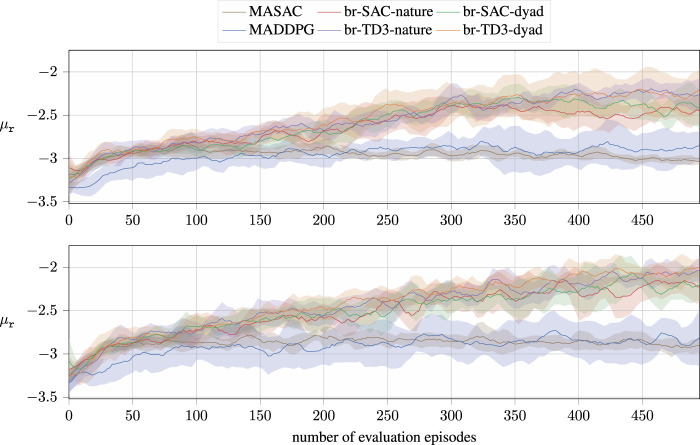
Results of the decentralized br-based algorithms for the cooperative collection task using sparse rewards. The figures present averaged rewards of all agents over eight learning runs per algorithm and environment. The shaded areas highlight a CI of 70%. The upper figure shows the performance of the collection task with static goal locations, whereas the environment on the bottom samples new goal locations upon every reset. The *x*-axis denotes the evaluation steps, which are run after 10 exploration episodes to evaluate the current performance.

For brevity, we present the evaluation performance of the individual algorithms based on the average rewards of all agents in Figure 3. In here, the term *evaluation* refers to the agents greedily following their current policies than drawing samples from them. The collected results show a static version, i.e., using fixed goal locations, in the lower figure and a non-static version in the upper figure. In this environment, three agents update their policies over 5,000 exploration episodes. The evaluation is only run every 10th episode, so the number of evaluation steps is lower than the actual explorations. In addition, the averaged reward per evaluation run is logged, which, in return, strongly depends on a randomly sampled starting state of the agent and the goal locations in the non-static environment. As a result, the collected data encounter high noise, which is reduced by smoothing the collected reward values using a Savitzky–Golay filter (Savitzky and Golay, 1964) and the implementation by Virtanen et al. (2020).

As it can be seen, our algorithms outperform the current state-of-the-art algorithms not only in terms of the final performance but also in terms of convergence speed for both scenarios. Unsurprisingly, our method performs best in static environments, requiring reaching static goal locations. In these scenarios, there is a direct relation between the agent states and the actual value functions, which leads to an improved learning speed.

For a closer evaluation of our presented algorithms, the per-agent rewards metrics are listed in Table 1. Furthermore, the number of total successful trials per algorithm during exploration and evaluation is listed. Exploration is not only run distinctly more often but also contains double the amount of steps per run. As a consequence, the number of successful exploration runs is distinctly higher compared to the evaluation numbers.

**TABLE 1:** Detailed performance metrics for evaluated environments. The results of the static environment are listed on the bottom. The values show the averaged results with the optional standard deviation appended by ±. The terms dyadic and nature are abbreviated by their first letter for brevity. Similarly, the number of successful trials of the exploration and evaluation runs are denoted as $d_{\text{ex}}$ and $d_{\text{ev}}$ .

	Static	MADDPG	MASAC	br-TD3-d	br-TD3-n	br-SAC-d	br-SAC-n
$r^{\left(1\right)}$		−2.92 ± 0.38	−2.95 ± 0.24	**−2.54 ± 0.48**	−2.55 ± 0.47	−2.6 ± 0.43	−2.64 ± 0.42
$r^{\left(2\right)}$		−2.94 ± 0.4	−2.96 ± 0.23	**−2.54 ± 0.48**	−2.57 ± 0.47	−2.6 ± 0.43	−2.64 ± 0.43
$r^{\left(3\right)}$		−2.99 ± 0.44	−2.95 ± 0.23	**−2.54 ± 0.48**	−2.55 ± 0.46	−2.61 ± 0.44	−2.64 ± 0.43
$d_{\text{ev}}$		3	0	**35**	31	12	10
$d_{\text{ex}}$		86	62	**1121**	901	256	246
$r^{\left(1\right)}$	✓	−2.89 ± 0.46	−2.87 ± 0.3	**−2.4 ± 0.51**	−2.41 ± 0.51	−2.5 ± 0.52	−2.51 ± 0.53
$r^{\left(2\right)}$	✓	−2.97 ± 0.5	−2.87 ± 0.3	**−2.42 ± 0.51**	−2.44 ± 0.52	−2.5 ± 0.52	−2.51 ± 0.53
$r^{\left(3\right)}$	✓	−2.88 ± 0.44	−2.87 ± 0.3	**−2.41 ± 0.51**	−2.44 ± 0.52	−2.5 ± 0.52	−2.52 ± 0.53
$d_{\text{ev}}$	✓	7	0	50	**52**	31	41
$d_{\text{ex}}$	✓	289	68	**1391**	1284	554	481

The best performing values are highlighted in bold.

Nonetheless, the collected numbers underline that our presented method outperforms current state-of-the-art methods distinctly, not only in terms of averaged accumulated rewards, as shown in Figure 3, but also for each individual agent involved. The performance increase becomes evident on comparing the success rates of the algorithms, where MASAC failed completely to find a successful policy.

Comparing the overall results, the TD3 agents outperformed not only the state-of-the-art methods but also our SAC variants. Furthermore, the dyadic setup resulted in improved performance for all evaluation metrics compared to the game-against-nature schematic representation. This confirms our initial statement that it is preferable to handle interactions individually rather than regressing interaction schemes fully from a NN.

Regarding the standard deviations of our proposed methods, it also becomes evident that our methods suffer from higher variance in the accumulated rewards. Even though this may seem like a disadvantage of our approach compared to the existing algorithms, it has to be noted that the obtained reward from a successful episode usually distinctly differs from the reward obtained from an unsuccessful episode. Therefore, the increased variance is not subject to our method but a consequence of the increased success rates, as highlighted in Table 1, where our algorithm achieves distinctly higher success rates than the existing AC methods.

In summary, it can be stated that our presented algorithm outperforms the existing methods within our simulated environments even though they are run fully decentralized.

## 6 Discussion and technical extensions

Given our presented algorithm, we conclude this article with an outline of possible extensions in order to further improve the overall performance.

### 6.1 Applying best-response policies on competitive environments

A current drawback of our algorithm is the restriction to competitive domains. If the agents have access to all reward values during learning, an additional critic for the objective of the other agent can be added to the presented algorithm. This results in applying the gradient step for the br policy not only over the joint task critic for the current agent but also the agent-specific agent critic. If this metric is applied, applying the dyadic interaction scheme from above is strongly recommended as our algorithm is restricted to optimizing the average reward over all agents otherwise.

Another extension is given by modeling non-cooperative agent(s). In order to model this procedure, it is best to condition non-cooperative agents on the joint team policy of all cooperative agents, thus leading to the conditional interaction policy as follows:

$$\begin{array}{cc} \underline{\pi }\left(\underline{s}\right)\approx & {\underline{\pi }}^{\left(-i,-i\right)}\left(s^{\left(i\right)}|{\underline{a}}^{\left(-i,i\right)},a^{\left(i\right)}\right){\underline{\pi }}^{\left(-i,i\right)}\left(s^{\left(i\right)}|a^{\left(i\right)}\right)\;{\underline{\pi }}^{\left(i\right)}\left(s^{\left(i\right)}\right), \end{array}$$ (19)

In Eq. 19 the cooperative policy is denoted as ${\underline{\pi }}^{\left(-i,i\right)}\left(s^{\left(i\right)}|a^{\left(i\right)}\right)$ and the non-cooperative policy is denoted as ${\underline{\pi }}^{\left(-i,-i\right)}\left(s^{\left(i\right)}|{\underline{a}}^{\left(-i,i\right)},a^{\left(i\right)}\right)$ . Alternatively, on-policy-based approaches, such as proximal policy optimization (PPO) or trust region policy optimization, are worth an investigation to model the behavior of other agents. Here, a direct approach is given by conditioning the policy estimate on the average over all estimators. A more promising approach would be given by averaging over all agent advantages and, thus, applying a gradient step. This bears the potential of stabilizing the estimated opponent models and, thus, the overall task-critic updates, which eventually increases the likelihood of converging to the team-optimal policy. Furthermore, there is great potential on incorporating the findings of Jaques et al. (2019) and, thus, not only regressing the agents’ policies but also directly conditioning the reward of each agent on the influencing reward.

### 6.2 Improving convergence behavior by partially centralized learning

The presented method fully decouples learning by learning opponent models without applying centralized learning schemes. This is endangered to leading to divergent agent behavior and thus converging to suboptimal team-behavior. Therefore, our current method could be further enhanced by introducing centralized learning without adding restrictive full observability assumptions. Rather than sharing the full observations, the individual opponent policy predictions can be shared during learning such that the policy gradient can be conditioned on the Kullback–Leibler (KL)-divergence of the predicted opponent policies.

$$\begin{array}{cc} a^{\left(j\right)} & \leftarrow {\pi^{\left(j\right)}}_{\Pi ,br}\left(s^{\left(i\right)},a^{\left(i\right)}\right)\\ J\left({\pi^{\left(j\right)}}_{\Pi ,br}\right) & =E_{s^{\left(i\right)},a^{\left(i\right)}}\left[{\left.\nabla_{a^{\left(-i\right)}}Q_{int}^{\left(i\right)}\left(s^{\left(i\right)},\underline{a}\right)\right|}_{a^{\left(j\right)}}-\frac{1}{N_{A}-2}\overset{N_{A}}{\underset{\begin{array}{c} k=1\\ k\neq j\\ k\neq i \end{array}}{\sum }}D_{\text{KL}}\left(\pi^{\left(j\right)}||\pi^{\left(k\right)}\right)\right] \end{array}.$$ (20)

Eventually, a baseline policy, e.g., obtained from behavioral cloning, can be substituted into Equation 20 to further stabilize the gradients.

### 6.3 Multi-robot hierarchical actor critic

Recalling the motivation of our algorithm in Section 4, a major advantage of our algorithm is the possibility of applying asynchronous actions. As this directly allows extending AC for MARL to HRL, we outline a conceptual extension of our approach tailored to RL tasks with sparse rewards. This extension relies on a collection of assumptions, which is also assumed in Levy et al. (2019):

• There exists an agent-specific state space $X^{\left(i\right)}\in S^{\left(i\right)}$ , and $x^{\left(i\right)}\in s^{\left(i\right)}$ always holds^1^.
• There exist deterministic mapping functions Fg:=X(i)×A(i){p}↦G(i){p−1}∈X(i) and Fg−1:=X(i)×G(i){p−1}↦A(i){p} , which map the actions of the upper layer to the goal space of the lower layer and *vice versa* for each agent.
• There exists a deterministic evaluation metric J{p}:=G×X(i){p}↦0,1 that evaluates the achievement of a goal g(i){p}, given the current agent state $X^{\left(i\right)}$ .

This differs from the original assumption by Levy et al. (2019) because we propose to explicitly distinguish between the internal agent state $x^{\left(i\right)}$ and the full environment observation $s^{\left(i\right)}$ .

We claim that within multi-agent HRL, it is specifically beneficial to distinguish between internal and external observations. Therefore, we propose to use structured observations as follows:

$$s^{\left(i\right)}:={\left(x,y_{e},y_{\left(-i\right)}\right)}_{i\in N_{A}},$$ (21)

In Eq. 21 $x^{\left(i\right)}$ reflects the internal state of an agent, e.g., current position, velocity, etc., and environmental observations $y_{e}^{\left(i\right)}$ from $A^{\left(i\right)}$ ’s perspective, e.g., images or laser range data, as well as observations of the other agents $y_{\left(-i\right)}^{\left(i\right)}$ .

Given this representation, we propose a two-layered hierarchy where the upper layer proposes sub-goals to the lower layer agents. This lower level is denoted as the *environment-layer* or p{e} in the following, while the upper layer is denoted as the *team-coordination* layer or p{i} . On the lowest layer, our decentralized MARL approach based on br policies from Section 4.1 can be applied using a dyadic interaction scheme.

Therefore, the individual components per agent are given as follows:

• A joint task critic $Q_{int}^{\left(i\right)}\left(s^{\left(i\right)},\underline{a}\right)$ .
• A native hierarchical critic $Q_{nat}^{\left(i\right)}\left(x^{\left(i\right)},y_{e}^{\left(i\right)},g^{\left(i\right)}\right)$ .
• A goal-conditioned action policy for the current agent $\pi^{\left(i\right)}\left(s^{\left(i\right)},g^{\left(i\right)}\right)$ .
• The dyadic br policies ${\left(\pi^{\left(j\right)}\left({\left(x,y_{e},y_{\left(-i\right)},a\right)}_{i\in N_{A}}\right)\right)}_{j\in -i}$ .

As the individual agents are provided with a sub-task that is to be reached by the dedicated agents alone, the hierarchical native critic preferably drops the dependency on the observation of other agents. In other words, this native hierarchical critic evaluates the hierarchically imposed rewards instead of estimating the current step-cost from the deviation with regard to the average reward. As a result, the update step of the lower layer follows Algorithm 1 but replaces the difference in line 9 by an average over the two critics. Furthermore, the native critic not only evaluates the environmental task success d but also evaluates if the update step has accomplished the current sub-goal. As the rest remains identical to Algorithm 1 and Equations 15 and 16, we omit repeating the same equations.

In contrast, the upper layer only tracks a single critic as infeasible sub-goals result in unpredictable task performance. Unfortunately, the agents do not have access to the goal mapping of other agents such that it is impossible to directly impose their policies or higher-level actions in the critic within decentralized settings. Furthermore, the upper layer usually suffers from asynchronous decisions, which would require adding the decision epochs to the critic’s state to allow sampling from the experience buffer. Therefore, we propose to apply an observation oracle instead of a br policy,

π{i}br(j):=X×Yenv×Y(j)×a{i}i∈NA↦Y(j), (22)

i.e., instead of predicting the agent action on the upper layer, the next observation is predicted. In case a (partially) centralized learning scheme is applied, this observation oracle can also be replaced with the following:

π{i}br(j):=X×Yenv×X(j)×a{i}i∈NA↦X(j), (23)

thus predicting the next internal state of the agent *j*. These opponent models from Eqs 22, 23 allow using data from an experience buffer independent of the higher-level policies or decision epochs during execution. As a result, the (interactive) task critic of the upper layers are regressed from Eqs 24 or 25:

Q{i}int(j):=S(i)×A(i)×Y(j)(i)j∈−i↦R (24)

Q{i}int(j):=s×a(i)×X(j)(i)j∈−i↦R (25)

Although the lower layer is updated similarly to Algorithm 1, the lower-layer critic update is given by calculating the following:

p=1T{i}max∑n=1T{i}maxγT{i}max-nr(i)st+n,a_t+n,st+n+1+1−d0if Jx(i)′,Fgx(i),a(i){i}↦⊤−Tmax−nif ∃n:Jx(i)t+n+1,Fgx(i),a(i){i}↦⊤−Tmaxelse+γ1−dmink=1,2Q{i}†int,ks(i)′,a(i)′{i},y(j)(i)′j∈−i(i), (26)

where T{e}max denotes the number of maximum sub-steps for a hierarchical transition and s′:=st+T{i}max represents the observation of agent *i* after a hierarchical update step. The actions and observations for the target critic in Equation 26 are obtained via the proposed policies from Eq. 27

a(i)′{i}←π(i){i}†Πs(i)′y′←π{i}†br(j)Ξs(i)′,a(i)′{i}j∈−i. (27)

The first term in Equation 26 averages the environmental reward, while the second adds the hierarchical penalty term depending on whether the lower layer could achieve the current action or the respective sub-goal. Eventually, the value function is approximated via querying the current higher-level policy and predicting the observations of the other agents.

## 7 Conclusion

Within this article, we have proposed a novel MARL framework that allows decentralized learning while differentiating between agent-based native costs and joint task rewards. In order to regress the team-optimal policy, each agent not only updates their own policy but also instead models the team-optimal response by estimating the br policy of the other agents. We propose to employ concepts from game theory, namely, either applying dyadic br policies for each agent pair or representing the collection of other agents as a game against nature. Even though our method relies on estimates of the agent-based costs, it outperforms recent state-of-the-art methods in terms of convergence speed within sparsely rewarded environments.

Given the promising results collected in simulation, this article provides a variety of extensions, which bear great potential for future research. First, an extension to competitive domains, i.e., zero-sum games, has been sketched by minimizing instead of maximizing the agent task critic when updating the other agents’ policies. Second, we outlined that sharing the predicted br policies can improve the convergence. Eventually, we sketched an extension of our method to a hierarchical MARL algorithm as this may allow bootstrapping performance during learning.

Using such a hierarchical MARL algorithm combined with structured environment observations bears the additional advantage of explicitly incorporating available model knowledge. This allows leveraging the concept of full end-to-end learning and instead combines MARL with optimization-based techniques, which remains a rarely covered field of research.

## Figures and Tables

**Table:** 

**Acronyms**	
**AC**	actor–critic
**br**	best response
**CI**	confidence interval
**COMA**	counterfactual multi-agent
**DDPG**	deep deterministic policy gradient
**DPG**	deterministic policy gradient
**DQN**	deep Q-network
**GPU**	graphics processing unit
**HRL**	hierarchical reinforcement learning
**KL**	Kullback–Leibler
**MAAC**	multi-actor-attention-critic
**MADDPG**	multi-agent deep deterministic policy gradient
**MARL**	multi-agent reinforcement learning
**MASAC**	multi-agent soft actor–critic
**MDP**	Markov decision process
**MG**	Markov game
**ML**	machine learning
**MPE**	multi-agent particle environment
**MPO**	maximum *a posteriori*policy optimization
**NE**	Nash equilibrium
**NN**	neural network
**PDF**	probability density function
**PG**	policy gradient
**PPO**	proximal policy optimization
**RL**	reinforcement learning
**SAC**	soft actor–critic
**SGD**	stochastic gradient descent
**TD3**	twin-delayed deep deterministic policy gradient
**TRPO**	trust region policy optimization
**Notation**	
p	placeholder variable in notation
***⊤***	Boolean *true*
**: =**	equal by definition
p{k}	hierarchical layer indexing
$1^{p\times p}$	identity matrix of dimension p×p
$0^{p\times p}$	zero matrix of dimension p×p
P	matrix in $R^{m\times n}$
R	rational numbers
***ζ***	threshold value; indexing defines actual meaning
t	current time or temporal indexing variable
$T_{\text{max}}$	maximum run time (continuous) or the number of time steps (discrete) in $R^{1}$
p	vector in $R^{n}$
$1^{p}$	identity vector of dimension p
$0^{p}$	zero vector of dimension p
**List of Symbols**	
a	action of the agent *i*
***π***	action assignment policy that maps a state to an action a
d	binary done flag, symbolizing the end of a task
r	single step reward return
$\pi_{\text{br}}^{\left(j\right)}$	dyadic best-response policy of the agent *j*to the action of the agent *i*
${\underline{\pi }}_{\text{br}}^{\left(-i\right)}$	dyadic best-response policy of agent *nature*to the action of agent *i*
***x***	Cartesian *x*-coordinate in $R^{1}$
***y***	Cartesian *y*-coordinate in $R^{1}$
v	damping constant
D	data buffer containing experiences usable for RL.
***α***	entropy temperature parameter for SAC
e	environment layer index
***f***	force magnitude or scalar force component in $R^{1}$
**Θ**	function approximation parameterization for a critic
**Π**	function approximation parameterization for a policy *π*
**Ξ**	function approximation parameterization for $\pi_{\text{br}}^{\left(j\right)}$ or ${\underline{\pi }}_{\text{br}}^{\left(-i\right)}$
i	interaction layer index
***D***_**KL**_	Kullback–Leibler-divergence of two PDFs
***ι***	learning rate for SGD
m	mass of a physical entity
**μ**	mean of a PDF.
$N_{A}$	number of agents
***v***	polyak-averaging weight, e.g., used to update the target network
A	player or (artificial) agent
s	state value in a general state space
x	fully observable state
y	hidden state
***γ***	temporal discount factor
***v***	translational velocity in SE (3)
**List of Operators**	
A	advantage function as the difference of Q and V in $R^{1}$
T	black box success function in $R^{1}$
R	black box reward function in $R^{1}$
***ρ***	PDF over a random variable
H	entropy of a random variable/PDF in $R^{1}$
E	expectation of a random variable/distribution
***χ***	general critic function in $R^{1}$ , e.g., V , A,or Q
$F_{g}$	goal mapping function; maps state/observation to a goal $g\in G\in R^{n}$
**∇**	gradient over a function or vector in $R^{n}$ or matrix in $R^{n\times n}$
L	loss function in $R^{1}$
J	general objective function for an optimization problem in $R^{1}$
P	probability of a random variable/distribution
Q	Q-function in $R^{1}$ , also known as state-action value function
***f***_**Π**_	deterministic function to apply the *reparameterization trick*, *cf.*SAC
T	stochastic transition function
V	value function in $R^{1}$
V **ar**	variance of a random variable/distribution V ar $\in R^{1}$

## Data Availability

The original contributions presented in the study are publicly available. This data can be found here: https://gitlab.com/vg_tum/multi-agentgym.git; https://gitlab.com/vg_tum/mahac_rl.git.

## Footnotes

1 Assumption of $x^{\left(i\right)}\in s^{\left(i\right)}$ emphasizes that we do not expect full state observability and that the intrinsic observations do not provide additional knowledge to the robotic agents.

## Appendix 1

The proposed algorithm has been evaluated on the MPE that has been extended from previous work (Lowe et al., 2017; Mordatch and Abbeel, 2017) to fit the scope of this article. The source code of the benchmark scenarios^2^ and the presented work^3^ can be found online, while the hyper-parameters and further implementation details leading to the results are listed in Appendix 2.

In order to meet the assumptions stated in Section 4, the original simulation environment has been adjusted such that the agents are able to differentiate between internal, external agent-related, and external environment-based observations. Thus, we introduce structured observations for our adjusted version of the MPE. Furthermore, the goal-mapping and evaluation metrics stated in Section 4 have been handcrafted and embedded into the dedicated environments similar to the original work of Levy et al. (2019). As claimed in Section 4, our approach tackles cooperative multi-robot RL tasks, such that only environments with pure continuous action spaces have been tested. Before outlining the experimental findings collected, we highlight the adjustments added to the default gym environment and the MPE.

### A.1 Structured observations in the multi-agent particle environment

In order to ease the implementation of the presented extensions in Section 6, our adjusted MPE directly allows obtaining the observation of each agent as $s^{\left(i\right)}:=\left(x^{\left(i\right)},y_{e}^{\left(i\right)},y_{\left(-i\right)}^{\left(i\right)}\right)$ . The MPE is characterized by $N_{A}$ agents moving in a *xy*-planar surface by applying a force on their body center. Thus, each agent is implemented as a point-mass, where the internal state and action are defined as

$$x^{\left(i\right)}:=\left[\begin{array}{c} x^{\left(i\right)}\\ y^{\left(i\right)}\\ {\dot{x}}^{\left(i\right)}\\ {\dot{y}}^{\left(i\right)} \end{array}\right]\;a^{\left(i\right)}:=\left[\begin{array}{c} f_{x}^{\left(i\right)}\\ f_{y}^{\left(i\right)} \end{array}\right],$$
(28)

where the action is a planar force^4^ actuated on the individual point-masses, which then follows the linear point-mass dynamics

$$x^{(i)\prime }:=\left[\begin{array}{cccc} 1 & 0 & dt & 0\\ 0 & 1 & 0 & dt\\ 0 & 0 & v & 0\\ 0 & 0 & 0 & v \end{array}\right]\left[\begin{array}{c} x^{\left(i\right)}\\ y^{\left(i\right)}\\ {\dot{x}}^{\left(i\right)}\\ {\dot{y}}^{\left(i\right)} \end{array}\right]+\left[\begin{array}{cc} 0 & 0\\ 0 & 0\\ \frac{1}{m} & 0\\ 0 & \frac{1}{m} \end{array}\right]a^{\left(i\right)},$$
(29)

using the mass of the entity m and a damping term $v\in \left[0,1\right]$ in free space. Each particle is set to a static size, i.e., a diameter of 0.05 m, which is used for collision checking with other agents or the environment. In case a particle collides with an object or an agent, a simple point-mass collision is applied. Even though the actual observation highly depends on the actual scenario or task to be solved, all our implementations contain the internal agent state in the observation of the agents.

As claimed in Section 4, our approach tackles cooperative multi-robot RL tasks in sparsely rewarded environments, such that only environments with pure continuous action spaces have been tested. In addition to *cooperative navigation*, we evaluated our approaches on the *cooperative collection* task, in which $N_{A}$ agents are expected to reach $N_{A}$ goal locations. The reward signal is provided sparsely, which is expressed as follows:

$$r^{\left(i\right)}\leftarrow \overset{N_{A}}{\underset{k=0}{\sum }}\left\{\begin{array}{cc} 0\; & \text{if }visited\left(g_{k}\right)\vee \exists j:{∥x^{\left(j\right)}-g_{k}∥}_{2}\leq \zeta_{g,MPE}\\ -1\; & \text{else}. \end{array}\right.$$
(30)

In addition, each agent is penalized with a direct cost value of −1 every time a collision with the environment or another agent is encountered. For both environments, the observations of other agents $y_{e}^{\left(i\right)}$ contain a two dimensional distance vector of the agents’ center position, while the observations of the environment $y_{\left(-i\right)}^{\left(i\right)}$ contain two-dimensional distance vectors to the goal locations and the objects in the environment.

## Appendix 2

The hyper-parameters for the learning procedure that is applied for all algorithms identically are listed in Table A1.

**TABLE A1:** Hyper-parameters for the experimental evaluation and all evaluated algorithms.

Parameter	Value
Batch size	1,024
Polyak value for the target net update	0.95
Decay parameter *γ*	0.95
Exploration episode length	200
Evaluation episode length	100
Number of episodes	5,000
Number of update steps	5
Update rate	every fifth episode
Episode buffer size	1,000
Number of agents $N_{A}$	3
Polynomial filter order Savitzky and Golay (1964)	3
Filter window size Savitzky and Golay (1964)	31

Similarly, the (physical) parameters for the MPE environment are listed in Table A2.

**TABLE A2:** Environment parameters for the MPE and *cooperative collection* task.

Parameter	Value
d*t*	0.02 s
$N_{A}$	3
Goal threshold *ζ*_*g*,MPE_ in Eq. 30	0.02 m
Collision cost	1
v	0.9
m	1 kg
***v***_max_	1.0 m/s
Number of collision objects	0

Eventually, the hyper-parameters for the individual algorithms are listed in Table A3. Our br-based policies used identical parameters for the dyadic and game-against-nature scheme. Therefore, only one column per algorithm is provided in the table below. We used Adam (Kingma and Ba, 2015) for the stochastic gradient descent (SGD)-optimization for all algorithms.

**TABLE A3:** Hyper-parameters for each algorithm. In case an entry is left blank, the algorithm does not have this hyper-parameter. The critic-parameters for our br-based approaches have been chosen identically for the interaction critic and native critic. Similarly, the parameters for the br policies are identical as the actor policy.

Parameter	MADDPG	MASAC	br-TD3	br-SAC
Size of (hidden) critic layers	(64, 64)	(64, 64)	(64, 64)	(64, 64)
Size of (hidden) policy layers	(64, 64)	(64, 64)	(64, 64)	(64, 64)
Learning rate critic $\iota_{Q}$	0.01	0.01	0.01	0.01
Learning rate policy *ι*_*π*_	0.01	0.01	0.01	0.01
Polyak value *υ*	0.01	0.01	0.01	0.01
*α*_0_		0.2		0.2

The experimental evidence was collected on two distributed computers with the following hardware components:

• **OS-Kernel**:
  • (Ubuntu) Linux-5.13.0–44
  • (Ubuntu) Linux-4.15.0–187
• **Processor**:
  • Intel(R) Core(TM) i3-7100 CPU @ 3.90 GHz
  • AMD Ryzen Threadripper 2990WX 32-Core
• **Python-version**: 3.9.7
• **GPU-acceleration**: disabled
